# Comparative Research on the Rebound Effect in Direct Electromagnetic Forming and Indirect Electromagnetic Forming with an Elastic Medium

**DOI:** 10.3390/ma11081450

**Published:** 2018-08-16

**Authors:** Xianlong Liu, Liang Huang, Hongliang Su, Fei Ma, Jianjun Li

**Affiliations:** 1 State Key Laboratory of Materials Processing and Die & Mould Technology, School of Materials Science and Engineering, Huazhong University of Science and Technology, Wuhan 430074, China; liuxianlong@hust.edu.cn (X.L.); sue@hust.edu.cn (H.S.); jianjun@hust.edu.cn (J.L.); 2 Changzheng Machinery Factory, China Aerospace Science and Technology Corporation, Chengdu 610100, China; mff126@126.com

**Keywords:** rebound effect, elastic medium, indirect electromagnetic forming, fittability

## Abstract

In the process of electromagnetic forming (EMF), the rebound effect caused by high speed collision between sheet and die will affect the fittability, which results in a bad forming quality of workpiece. In this paper, finite element models of direct EMF and indirect EMF with an elastic medium are established, the influence factors of fittability in indirect EMF are studied, the two forming processes are compared, and the mechanisms of reduced rebound effect in indirect EMF are revealed. The results show that: in indirect EMF, with the increase of the discharging voltage or thickness of rubber, the fittability increases and then decreases; when the thickness of driver plate is equal to the skin depth of the driver plate, the fittability is the best. The optimal process parameters of indirect EMF are as follows: the discharging voltage is 10 kV, the thickness of the rubber is 20 mm and the thickness of driver plate is 2 mm. The rebound effect in indirect EMF is reduced compared with direct EMF for the following reasons: the impact force caused by the collision between the sheet and die is balanced by the pressure provided by the rubber; the sheet is always under tensile stress state due to the friction force provided by rubber; the remaining kinetic energy of sheet after collision with the die is absorbed by rubber. Therefore, the rebound effect in indirect EMF is suppressed compared with direct EMF. So, the fittability of the workpiece is improved, which results in a better forming quality.

## 1. Introduction

The forming limit of aluminum alloy under a traditional quasi-static stamping forming condition is low, so fracture defects occur easily. The elastic modulus of aluminum alloy is small, so the springback problem is hard to control under a traditional quasi-static stamping forming condition, which results in lower fittability and bad forming quality of the deformed workpiece. Moreover, a press machine is needed in traditional quasi-static stamping forming [1,2]. Electromagnetic forming (EMF) is a high speed forming method, which can improve the formability limit of aluminum alloy sheets [3], and reduce the springback problem [4,5]. Furthermore, the EMF process has high forming efficiency, and it is not in need of lubrication and punch die [6]. As EMF has more advantages than traditional quasi-static stamping in forming aluminum alloys, it has been widely used in aviation, aerospace, and automobile fields [7].

The EMF of aluminum alloy sheet metal can be divided into two categories: free EMF and EMF with the die. At present, research on EMF with the die is mainly focused on improving the forming limit that is caused by the high speed collision between the sheet and die. Fenton et al. [8] simulated the impact pressure of EMF with the die, and found that the peak value of the collision pressure was about three times of the yield strength of the sheet material. Seth et al. [9] took electromagnetic ring expansion experiments with the die and without the die, and found that the number of fractures could be significantly reduced in not freely expanded rings, compared with freely expanded rings at the same strain level. Free forming and conical die experiments were carried out on aluminum sheets by Imbert et al. [10]. The results showed that the forming limit in the conical die condition was higher than the electromagnetic free bulging condition, because of the tool-sheet interaction on damage evolution. Metallographic observation was done by Imbert et al. [11], and it was found that the area porosity was smaller in forming with the die compared with the free bulging of the same strain condition. The above research shows that the forming limit of sheet metal in EMF with the die is increased compared with free EMF, but the rebound effect occurs in EMF with die because of the high speed collision between the sheet and die [12,13]. 

The rebound effect is not helpful for improving the dimension accuracy and the forming quality of EMF with the die. Bad dimension accuracy could lead to crevice corrosion and pitting that reduces the service life of potential products in adverse conditions [14]. Therefore, many scholars have done a lot of work on how to restrain the rebound effect in EMF with the die. Shim et al. [15] carried out EMF experiments on sheets using two different forming coils, and it was found that the helical-type coil was better than the bar-type coil, to reduce the rebound effect in formation. Risch et al. [16] simulated the EMF process of adding spring damping system at the bottom of the die. The results showed that with the increase of the damping coefficient, the rebound effect was decreased first and then it increased, and it was found that lower stiffness was helpful to achieve increased fittability. Based on the research of Risch, Liu et al. [17] proposed an improved spring damping system to reduce the rebound effect in EMF. Guo et al. [18] found that the fittability of the sheet is increased when two times discharging was applied in the same position compared with the single discharging. Yu et al. [19] presented that the fittability of the tube increased first and then decreased with the increase of discharging voltage in experiments of EMF of tube bulging. Su et al. [20] proposed two-step EMF to deform the sheet, and the results showed that improved forming accuracy was achieved over the single-step process because the rebound effect is reduced in the second step forming process. Noh et al. [21] studied the forming process of EMF with a cushion plate. It was presented that the deformed sheet was closely fitted with the objective die compared with the results of EMF without a cushion plate; this was due to the reduced rebound effect in EMF with a cushion plate. The above analysis shows that the existing research on suppressing the rebound effect in EMF are as follows: changing the coil structure, adjusting the discharging energy, optimizing the die structure, increasing the number of dischargings, and increasing the cushion plate. However, the research on effect of increasing elastic medium on the rebound effect in EMF is less.

Because the elastic medium such as rubber has the characteristic of absorbing energy, it is often used to cushion the impact force in the high-speed impact, and then to eliminate the fittability of the sheet metal and die after the collision. Li et al. [22] found that when an elastic medium was added in electro-hydraulic forming, the fittability of the deformed sheet was improved. Ezra et al. [23] found that it was helpful to reduce the impact force and to improve the forming quality in explosive forming by adding elastic medium of rubber. FEM simulation of laser forming process with rubber was done by Wang [24], and the results showed that the appropriate rubber thickness and low rubber hardness was helpful to eliminate the rebound effect. The above results show that the increase of the elastic medium in the process of explosive forming, electro-hydraulic forming, and laser forming is helpful to reduce the rebound effect in the forming process, and to increase the fittability of the deformed sheet, but research on the effect of increasing the elastic medium on the rebound effect of EMF is lacking. Moreover, there are few studies on the influence of factors on the fittability of the indirect EMF with elastic medium, and the comparative study on the indirect EMF with elastic medium and the direct EMF is lacking.

Therefore, the effect of adding an elastic medium on the rebound effect in the EMF process is studied by comparison of the experiments of direct EMF and indirect EMF in this paper. The influence of the discharging voltage, the thickness of the rubber, and the thickness of the driver plate on the fittability of the deformed sheet metal in indirect EMF are studied. Then, the process of direct EMF and indirect EMF are compared, and the reason for the reduced rebound effect of the deformed workpiece in indirect EMF is revealed.

## 2. Research Methods

### 2.1. Experiment Setup

**Figure 1:**
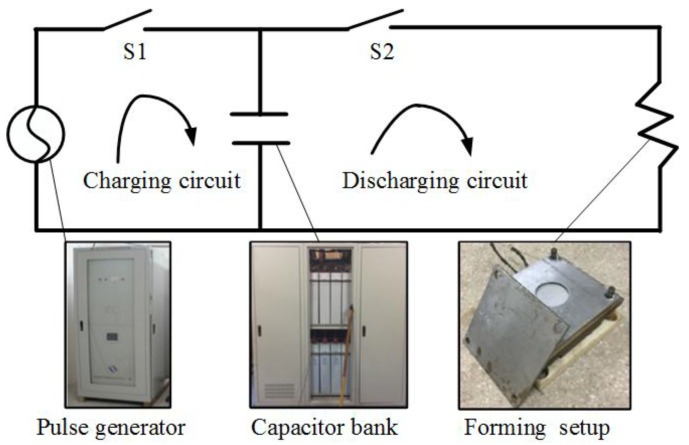
The EMF system for the experiments.

Figure 1 shows the EMF system used in the experiments. The EMF system contained a charging circuit and discharging circuit. During the charging process, the switch S1 is closed and S2 is opened. The discharging parameters are set by the software of EMF system. The discharging parameters are then transmitted to the HMF-30/213-150 type electromagnetic pulse generator (National High Magnetic Field Center, Wuhan, China) to control the charging process. When the energy in the capacitor bank is filled, the charging process is ended. Then, the switch S1 is opened and S2 is closed, and the discharging process is begun. During the discharging process, the energy is transferred from capacitor bank to the forming setup, to apply the EMF experiment. The capacitor bank consists of sixteen small capacitors, and the total capacitance is 212.8 μF. The maximum discharging voltage of the setup is 30 kV, the maximum discharging current is 150 kA, and the highest discharging energy is 95.76 kJ.

**Figure 2:**
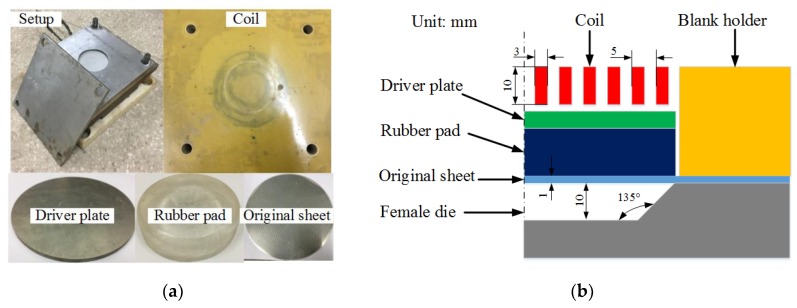
Forming setup of indirect EMF (**a**), and configuration of the setup (**b**).

The setup of the indirect EMF is shown in Figure 2a, and the configuration of the setup is shown in Figure 2b. The diameter of the die is 100 mm, the forming angle is 45 degrees, and the forming depth is 10 mm. The forming coil is a flat spiral coil, the coil section is 3 mm × 10 mm, and the turn spacing is 2 mm. The inner diameter of the coil is 18 mm and the outer diameter is 48 mm. The forming coil is made by the following processes: firstly, the spiral groove is machined on the epoxy board; secondly, the copper wire is wounded into the groove; thirdly, the copper wire is bonded with the epoxy board by epoxy adhesive; finally, a thin epoxy plate is glued to the surface. The material of the sheet is 5052 aluminum alloy. The diameter of the sheet is 180 mm and the thickness is 1 mm.

### 2.2. Establishment of Simulation Models

**Figure 3:**
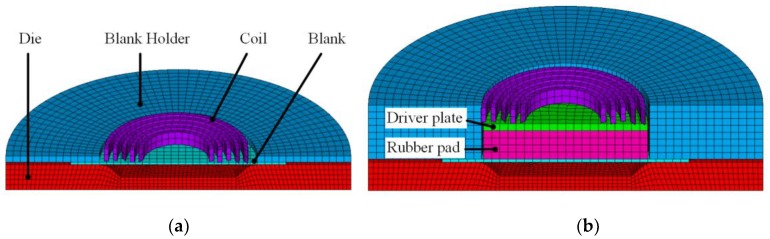
Model of direct EMF (**a**) and Model of indirect EMF (**b**).

The steps of establishing the models in this paper were as follows: at first, the geometry models of direct EMF and indirect EMF were built in UG NX8 (SIEMENS, Berlin, Germany), secondly, the mesh files were generated by ANSYS command flows (ANSYS, Pittsburgh, PA, USA), thirdly, the mesh files were modified in LS-PREPOST (LSTC, Livermore, CA, USA) to add the electromagnetic boundary conditions, then the simulations were done by the use of the EM module in LSDYNA (LSTC, Livermore, CA, USA), and finally the results are analyzed in LS-PREPOST (LSTC, Livermore, CA, USA). The established finite element model of direct EMF is shown as Figure 3a. Figure 3b shows the finite element model for indirect EMF. In order to improve the precision of the models, hexahedral meshes were used for all the parts in the models. Compared with direct EMF, the driver plate and the elastic medium of rubber were added in indirect EMF. The material of the driver plate was the same as that of the sheet, which is annealed 5052 aluminum alloy. The elastic medium was selected to be rubber with a hardness of 70 ShA. The material model of the rubber is Mooney-Rivlin model, which can be expressed as Equation (1):

(1) $$W=\sum_{k+m=1}^{n}C_{\mathrm{km}}{\left(I_{1}-3\right)}^{k}+{\left(I_{2}-3\right)}^{m}+\frac{1}{2}k{\left(I_{3}-1\right)}^{2},$$

in which, $I_{1}$, $I_{2}$, and $I_{3}$ are strain variables, and k is the elastic modulus. Usually two Mooney-Rivlin parameters ($C_{10}$ and $C_{01}$) are used to describe the superplastic deformation behavior of rubber. The parameter used in this paper is $C_{10}$ = 0.736, $C_{01}$ = 0.184 [24].

**Figure 4:**
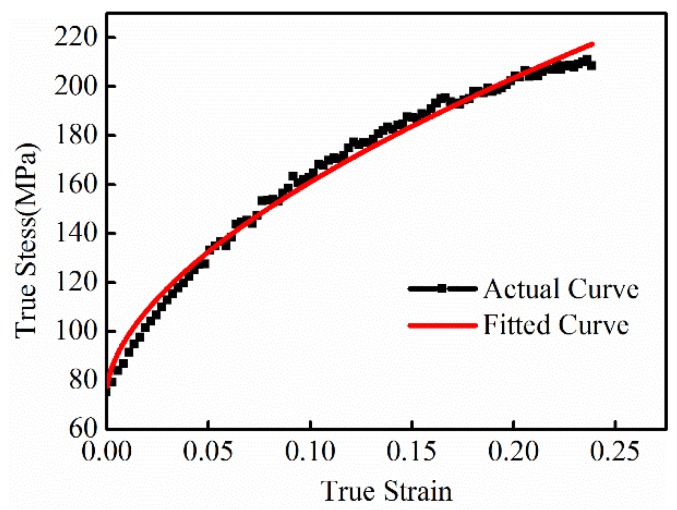
Quasi-static true stress-true strain curve of AA5052.

In order to consider the effect of the high strain rate on the forming process, the viscoplastic material behavior with rate-dependence law (Cowper–Symonds constitutive model) was used to study the effect of high strain rate on the forming process, as shown in Equation (2):

(2) $$\sigma =\sigma_{0}\left(1+{\left(\frac{\dot{\varepsilon }}{P}\right)}^{m}\right).$$

in which, $\sigma_{0}$ is dynamic flow stress, $\dot{\varepsilon }$ is plastic strain rate, and *p* and *m* are material parameters. For 5052 aluminum alloy, *p* = 6500 s-1 and m = 0.25 [25]. The constitutive relation of 5052 aluminum alloy for quasi-static forming was achieved from an Instron electrical tensile testing machine, which is shown in Figure 4. The current trace was set as the input load for the simulation, which can be expressed by Equation (3):

(3) $$I=U\sqrt{\frac{C}{L}}\exp\left(-\frac{Rt}{2L}\right)\sin\left(\frac{1}{\sqrt{LC}}t\right).$$

**Figure 5:**
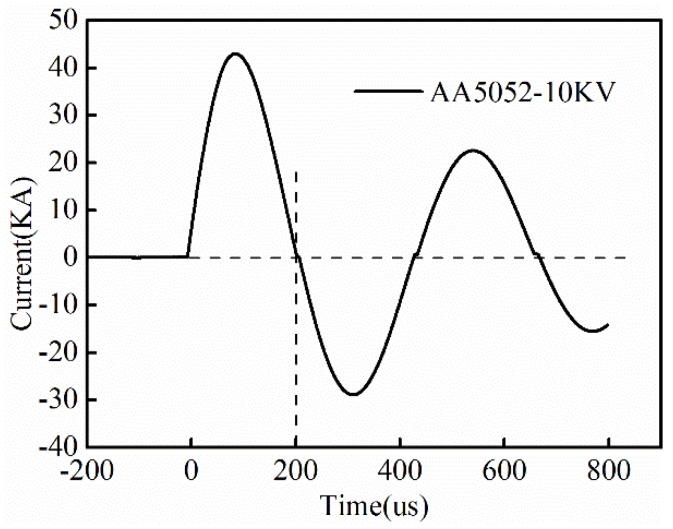
Measured coil current under a discharging voltage of 10 kV.

The current trace of a 10 kV energy input case was measured by a Rogovoski coil, which is shown in Figure 5. Then, the parameters in Equation (3) were computed as follows: capacity *C* is 212.8 μF, inductance *L* is 88.2 μH, and resistance *R* is 83 mΩ. As the workpiece moved away from the coil quickly, the following current after the first half has little effect on the forming process. Therefore, only the first half cycle of the current curve was extracted as the coil load in the simulation [26]. In addition, the friction factor between the sheet and die was selected as 0.15 [27]. To consider the restoring force of the rubber, the model for rubber elasticity was selected as the freely-jointed chain model, and the worm-like chain model [28].

### 2.3. Validation of Simulation Models

**Figure 6:**
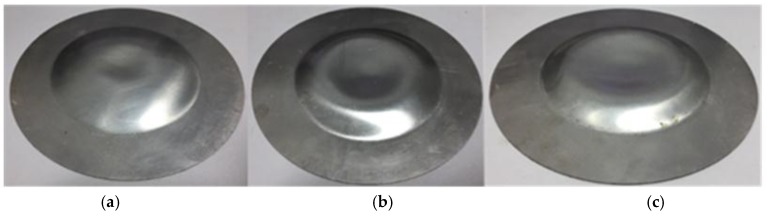
Deformed workpieces of direct EMF under different discharging voltages (**a**) 6 kV; (**b**) 8 kV; (**c**) 10 kV.

The workpieces after direct EMF under different discharging voltages are shown in Figure 6. When the discharging voltage was 6 kV, it can be seen from Figure 6a that there was a pit at the center of the sheet metal after direct EMF, and the sheet could not be fitted well with the die. This is because of the rebound defects caused by the impact between the sheet and die. As can be seen from Figure 6b, when the discharging voltage was 8 kV, the diameter and depth of the pit at sheet center increased compared with the workpiece under a discharging voltage of 6 kV. When the discharging voltage increases to 10 kV, the pit’s diameter was larger, and the depth was deeper, which is shown in Figure 6c. This is because the increasing discharging voltage resulted in increasing collision speed between the sheet and die. So, a higher impact pressure was generated, and this is why the rebound effect was more obvious.

**Figure 7:**
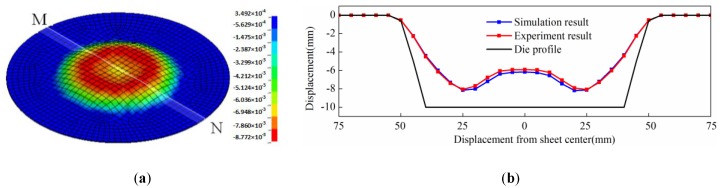
Simulation result of direct EMF (**a**) and comparison results of profile data along MN (**b**).

When the discharging voltage was 10 kV, the simulation result of direct EMF is shown in Figure 7a. It can be seen that the rebound effect occurred at the sheet center after direct EMF, and the shape of the workpiece was in agreement with the experimental result, as shown in Figure 6c. MN was through the sheet center, and it was on the lower surface of the sheet which was in contact with the female die during forming. The profile data along MN of simulation results was taken out and compared with the data obtained from the experimental results. The comparison of simulation result and experimental result is shown in Figure 7b. It was shown that the maximum deviation was 1.5%. It can be found that fittability of simulation result was a little better than the experiment result. The reason may be that air resistance was neglected in the simulation process. The results showed that the simulation model could effectively predict the process of direct EMF, and that it was reliable and applicable.

**Figure 8:**
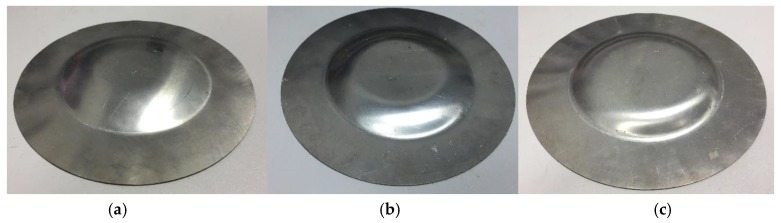
Deformed workpieces of indirect EMF under different discharging voltages (**a**) 6 kV; (**b**) 8 kV; (**c**) 10 kV.

The workpieces after indirect EMF under different discharging voltages are shown in Figure 8. It can be seen from Figure 8a that when the discharging voltage was 6 kV, the bottom of the deformed workpiece was flat after indirect EMF. Compared with the result after direct EMF, which is shown in Figure 6a, there was no pit defect at the sheet center. It was indicated that the rebound effect caused by the collision between the sheet and the die was inhibited, and the addition of rubber and driver plate was helpful to improve the fittability of deformed sheet. When the discharging voltage was 8 kV, the deformed workpiece after indirect EMF is as shown in Figure 8b. It can be seen that the flat area of the sheet bottom was increased, compared with the condition of 6 kV. This is to say the fittability was better. Figure 8c shows the deformed workpiece of 10 kV discharging voltage, and it was found that the flat area of the bottom of the sheet was increased with an increasing discharging voltage. The above results show that the rebound effect could be suppressed in indirect EMF with a rubber and driver plate. 

**Figure 9:**
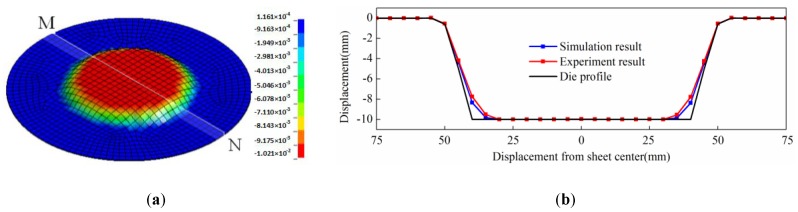
Simulation result of indirect EMF (**a**) and Comparison results of profile data along MN (**b**).

When the discharging voltage is 10 kV, the simulation result of indirect EMF with elastic medium is shown in Figure 9a. It can be seen that the rebound effect is inhibited, which was the same as the experimental results, as shown in Figure 8c. The profile data along MN of simulation results is also taken out and compared with the data obtained from experimental results. The comparison of the simulation results and the experimental results are shown in Figure 9b, and it was shown that the maximum deviation was 1.3%. It can be found that fittability of the simulation result was a little better than experiment result. The reason may be that the air resistance was neglected in the simulation process. The results show that the simulation model can effectively predict the process of indirect EMF, and it is reliable and applicable.

## 3. Results and Discussions

### 3.1. Factors Affecting the Fittability of Indirect EMF

#### 3.1.1. The Orthogonal Experiments

Many forming parameters have great effect on the rebound effect in indirect EMF. Among these factors, the discharging voltage, the thickness of the driver plate and the thickness of rubber are chosen bases on preliminary research. In this paper, the orthogonal experimental method is used to evaluate and optimize the above three influencing factors by using the established finite element model. According to the preliminary research results, each parameter was selected out of four levels. The factors and levels of the orthogonal experiments are shown in Table 1. It can be seen that the discharging voltage was selected as 6, 8, 10, and 12 kV. The thickness of the driver plate was selected as 1, 2, 3, and 4 mm. The thickness of rubber pad is selected as 10, 20, 30, and 40 mm.

In the study, the simulated fittability η of the deformed workpiece was selected as the evaluation index. The value of η was defined as the mean distance deviation between the deformed workpiece and the die. η was computed by Equation (4) as follows:

(4) $$\eta =\left(1-\frac{\sum_{1}^{n}\left(g\left(x_{i}\right)-f\left(x_{i}\right)\right)}{n\cdot h}\right)\times 100\%.$$

**Figure 10:**
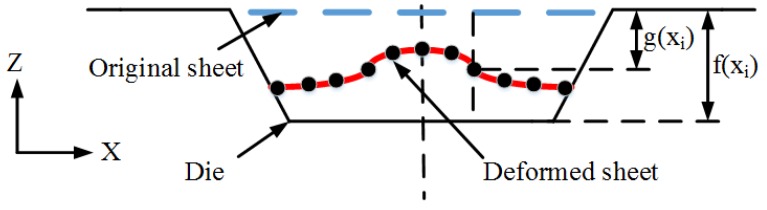
Schematic diagram of fittability.

As shown in Figure 10, the interval of selecting node $x_{i}$ on the sheet surface near to the die was 5 mm, $f\left(x_{i}\right)$ was the z-direction displacement of $x_{i}$, and g$\left(x_{i}\right)$ was the corresponding z-axis coordinate on the die, *h* was the forming depth of the die. When the η value was smaller, the fitting degree of deformed sheet and die was worse; when the η value was larger, the fitting degree of deformed sheet was better.

According to the conditions of three-factor four-level orthogonal test tables, the corresponding fittability of indirect EMF was simulated, as shown in Table 2. The fittability under the condition of each factor of each level was summed. The values were then divided by four to obtain the average value of fittability under the condition of each factor of each level. The average value of fittabilities of different discharging voltages were: 53.2%, 67.1%, 88.3%, and 81.1%. The average value of the fittabilities of different thicknesses of the driver plate were: 81.3%, 87.2%, 69.4%, and 65.1%. The average value of fittabilities of the different rubber thicknesses were: 82.2%, 86.6%, 65.3% and 62.1%. The range of the discharging voltage, the thickness of the driver plate and the rubber thickness is: 35.1%, 22.1% and 24.5% respectively. According to the results of range analyses, the influence of discharging voltage on the fittability of indirect EMF of 5052 aluminum alloy sheet was the largest among the three parameters. Rubber thickness was the second largest influence factor, and the thickness of the driver plate was ranked third.

#### 3.1.2. Effect of Discharging Voltage

**Figure 11:**
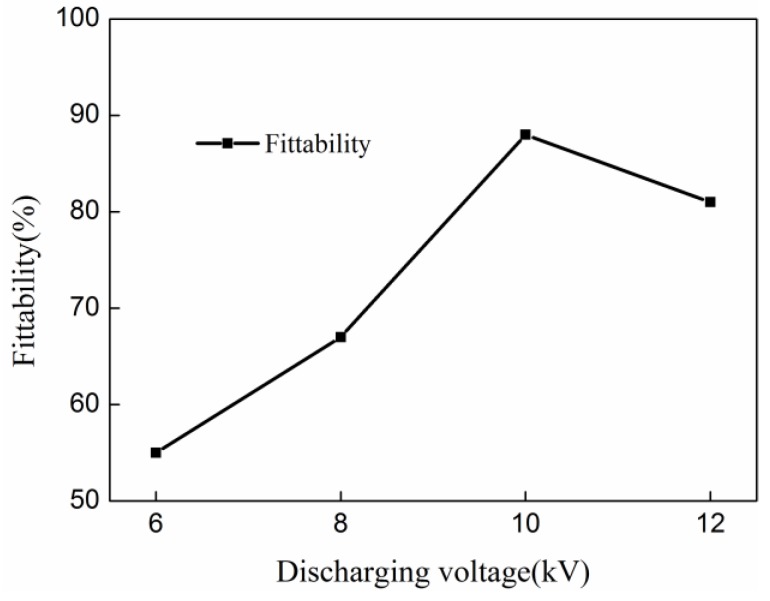
The influence of discharging voltage on the fittability of indirect EMF

Figure 11 shows the effect of discharging voltage on the fittability of the deformed sheet after indirect EMF. It can be seen that when the discharging voltage increases from 6 kV to 10 kV, the fittability was increased from 55.4% to 88.3%. This is because that if the discharging voltage was too small, the sheet had no contact with the die bottom after forming. This is to say, the deformation was not sufficient. However, when the discharging voltage increases to 12 kV, the fittability decreased to 81.2%. This is because that if the discharging voltage was too large, the kinetic energy transferred to the sheet is increased, which results in higher impact speed. As only part of the kinetic energy of the sheet could be absorbed by the rubber pad, the remaining kinetic energy on the sheet was caused the rebound effect. As a result, the optimal discharging voltage was 10 kV in this research.

#### 3.1.3. Effect of Thickness of Rubber Pad

**Figure 12:**
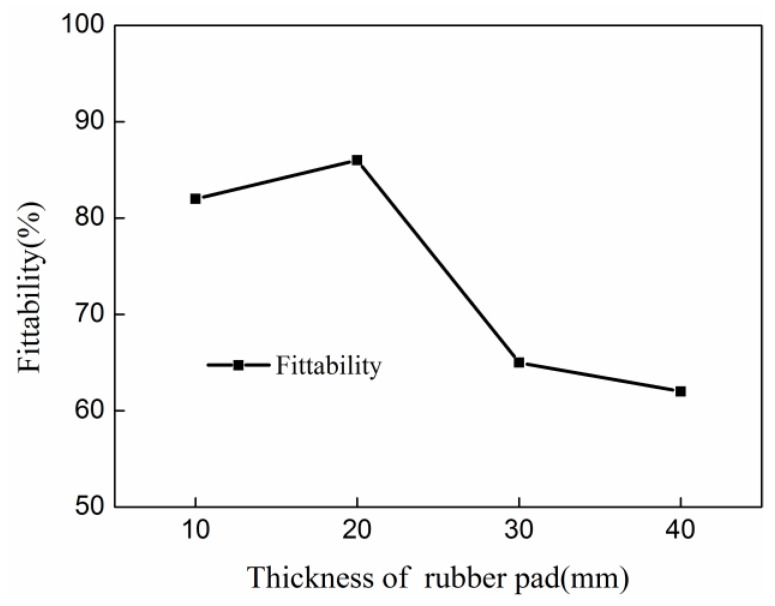
The influence of rubber thickness on fittability in indirect EMF.

Figure 12 shows the effect of rubber thickness on the fittability of deformed sheets after indirect EMF. It can be seen that when the thickness of rubber increased, the fittability of deformed sheet decreases first and then it increased. With a rubber thickness of 10 mm, the rubber is compressed excessively when the sheet is impacted with the die. It results in reduced ability of absorbing energy, so rebound effect is occurred as the residual kinetic energy of the sheet after collision cannot be fully absorbed. On the other side, the rubber played a role of force-transmitting medium in indirect EMF. This was the same of the water’s action in EHF [29]. If the rubber’s thickness was too small, the forces acting on the sheet were the same as direct EMF. As forces acting on different locations on the sheet were not the same, this would contribute to the generation of rebound effect. This was why that when rubber thickness was too small, lower fittability would be achieved. When the rubber thickness was 20 mm, the fittability was largest which equaled 87.3%. If the rubber thickness exceeded more than 20 mm, the fittability decreased with increasing rubber thickness. This is because that the total discharging energy was certain, and the energy expended on the rubber increased with increasing rubber thickness. It resulted in a lack of energy for sheet deformation, which led to a lower fittability of the deformed sheet. Therefore, the optimum thickness of rubber in this study was 20 mm.

#### 3.1.4. Effect of the Thickness of the Driver Plate

**Figure 13:**
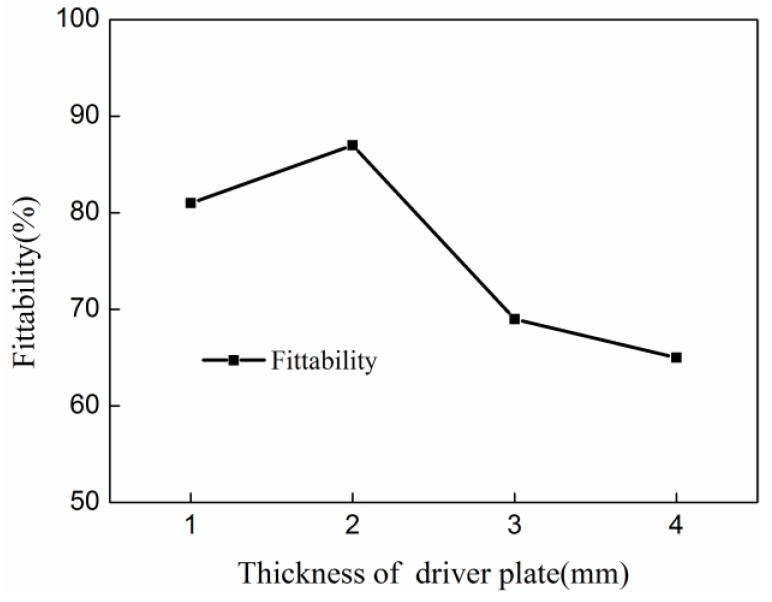
The influence of the thickness of the driver sheet on fittability in indirect EMF.

Figure 13 shows the effect of thickness of the driver plate on fittability of deformed sheet after indirect EMF. With the thickness of the driver plate increases, the fittability decreases first and then increases. When the thickness of the driver plate is 2 mm, the fittability is the largest. This is related to the energy efficiency of the driver plate. In EMF, the energy utilization was the highest when the thickness of the sheet was equal to the skin depth of the sheet material [30]. The skin depth of the sheet was shown in Equation (5):

(5) $$\delta =\sqrt{\frac{2}{\omega \mu_{0}\gamma }}.$$

in which *ω* is the angular frequency of the discharging circuit, $\mu_{0}$ is the permeability, and *γ* is the resistivity of the sheet. Thus, the skin depth of the 5052 aluminum alloy was about 2 mm under the condition of this study. Therefore, when the thickness of the driver plate was 2 mm, the energy utilization of indirect EMF was optimal. When the thickness of the driver plate does not exceed the skin depth, the magnetic field energy is lost through the sheet, resulting in a waste of energy. When the thickness of the driver plate exceeds the skin depth, the energy consumed on the deformation of the driver plate increases with increasing driver plate thickness. This results in energy reduction which is transferred to the sheet, which causes a lower fittability of the deformed sheet. Therefore, when the thickness of the driver plate is equal to the skin depth of 2.0 mm, the fittability is optimal.

### 3.2. Comparative Analysis of Indirect EMF and Direct EMF

The above research shows that the optimal process parameters for indirect EMF with the elastic medium are as follows: the discharging voltage was 10 kV, the thickness of the rubber was 20 mm and the thickness of the driver plate was 2 mm. In order to analyze the reason of the fittability improvement of the deformed workpiece after indirect EMF, the results of indirect EMF and direct EMF are compared and analyzed in this section. Indirect EMF was performed under the conditions of the combination of optimal process parameters, and direct EMF was performed under the conditions of the discharging voltage of 10 kV.

#### 3.2.1. Comparison of the Forming Process

**Figure 14:**
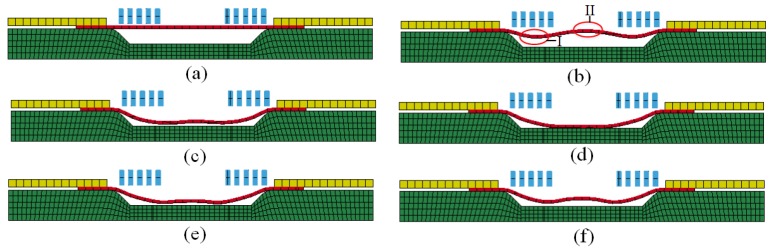
Deformation process of direct EMF (**a**) t = 0 μs; (**b**) t = 100 μs; (**c**) t = 200 μs; (**d**) t = 300 μs; (**e**) t = 400 μs; (**f**) t = 500 μs.

The deformation process of direct EMF under a discharging voltage of 10 kV is shown in Figure 14. As shown in Figure 14b, region Ι corresponded to half of the coil radius where the electromagnetic force was largest. Region ΙΙ corresponded to the center of the sheet, where the electromagnetic force was zero. When the forming time was 100 μs, the electromagnetic force reached its peak. Therefore, the material of area Ι was deformed first, which led to the deformation of region ΙΙ. When the forming time 200 μs, the action of electromagnetic force was to the end, and the deformation of sheet continued due to the action of the inertia force, as shown in Figure 14c. It can be found from Figure 14d that when the forming time is 300 μs, the material of region Ι is in contact with the female die, but the material of region ΙΙ was not impacted with the die. As shown in Figure 14e, when the forming time was 400 μs, the material of region ΙΙ collided with the die. It was found that reverse deformation happened first at region ΙΙ, which led to the reverse deformation of region Ι. Figure 14f shows that when the forming time was 500 μs, the deformation of the sheet was to the end, and a pit was generated at the center of the sheet, which is named the rebound effect.

**Figure 15:**
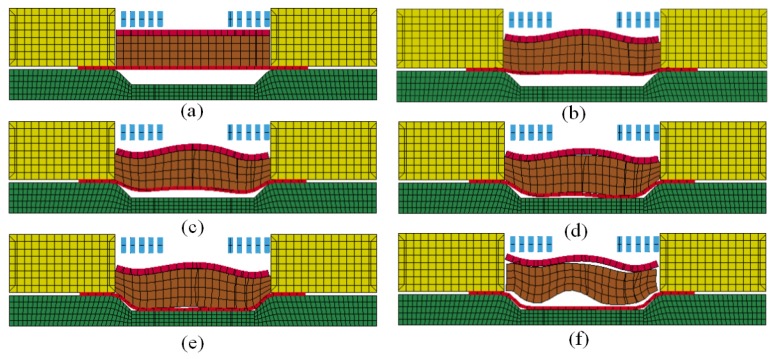
Deformation process of indirect EMF (**a**) t = 0 μs; (**b**) t = 100 μs; (**c**) t = 200 μs; (**d**) t = 300 μs; (**e**) t = 400 μs; (**f**) t = 500 μs.

The forming process of indirect EMF under the combination of the optimal process parameters is shown in Figure 15. It can be seen from Figure 15b that when the forming time was 100 μs, the deformation of driver plate was triggered by the action of the electromagnetic force. Then, the rubber was compressed by the action of the drive plate. When the pressure from the rubber exceeded the yield limit of the sheet, the plastic deformation of sheet was triggered. Compared with direct EMF, plastic deformation of different parts of the sheet in indirect EMF occurred almost simultaneously. This is due to the sheet being under uniform surface force from the elastic medium during indirect EMF, and the deformation velocity of different positions on the sheet being uniform. In the process of direct EMF, the sheet material is subjected to a Lorentz force, which is an uneven body force, so that the deformation velocity of different positions on the sheet are different. As shown in Figure 15c, when the forming time was 200 μs, the rubber was compressed continually, and the sheet was gradually deformed deeper. When the forming time was 300 μs, the sheet metal gradually came into contact with the die, as shown in Figure 15d. It was found that the center area of the sheet fitted well with the die, but the fittability at the die corner was not good. When the forming time was 400 μs, the rubber was compressed further, which results in an increased fittability of the workpiece, as shown in the Figure 15e. It can be seen that there was still a gap between the sheet and the die at the die corner; this was because it was difficult for the material in this area to flow to the die corner. Figure 15f shows that when the forming time was 500 μs, the rubber was separated from the sheet due to the elastic response of the rubber, and the sheet was fitted well with the die after separation with the rubber. By means of Equation (4), the fittability of the deformed workpiece was 60.1% under the condition of direct EMF. Under the condition of indirect EMF, the fittability of the deformed workpiece was 87.3%, which was 27.2% higher than that of direct EMF. It was indicated that the rebound effect was restrained in indirect EMF compared with direct EMF, and the addition of the rubber pad and the driver plate was helpful to improve the fittability of the deformed workpiece.

#### 3.2.2. Comparison of Displacement of Key Points on Sheet

**Figure 16:**
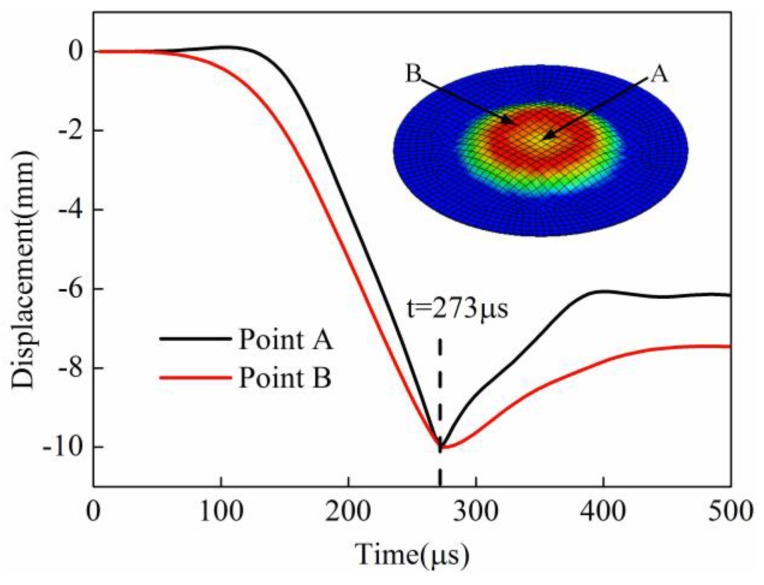
Displacement of point A and point B in direct EMF.

Point A and point B were selected to study the displacement change for direct EMF and indirect EMF. Point A corresponded to the sheet center, and point B corresponded to the position at half of the coil radius. Both of the two points were on the lower surface of the sheet, which was in contact with the die during forming. The displacement of point A and point B for direct EMF are shown in Figure 16. It can be found that the displacement of point A and point B gradually decreased with time, and then it increased after the collision between the sheet and die. The deformation of point B was prior to point A before 273 μs; this was because the magnetic force of point B was greater than that of point A. When the forming time was 273 μs, the displacement of point B and point A reached −10.0 mm successively. The displacement of these two points was reversed later, which indicated that both of the two points collided with the die. The final displacement of point A was about 6.2 mm, and the final displacement of point B was about 7.5 mm.

**Figure 17:**
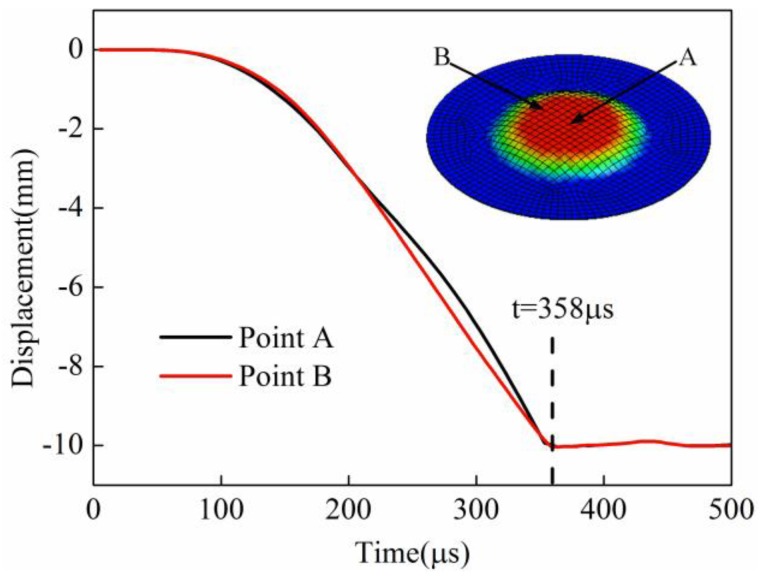
Displacement of point A and point B in indirect EMF.

The displacement of point A and point B for indirect EMF are shown in Figure 17. It can be seen that the displacement of point A and point B were basically coincident before 200 μs. The displacement of point B was slightly greater than point A between 200 μs and 300 μs. After 358 μs, the displacement of the two points were basically unchanged. Finally, displacement of point A and point B was equal to 10.0 mm, which showed that the two points were fitted well with the die. The above results showed that the displacement of point A and point B was not reversed in indirect EMF, that is to say, the rebound effect did not occur after the collision between the sheet and the die. From the comparison of the displacement of point A and point B for direct EMF and indirect EMF, it can be concluded that the rebound effect was effectively suppressed by adding the driver plate and the rubber pad.

#### 3.2.3. Comparison of Velocity of Key Points

**Figure 18:**
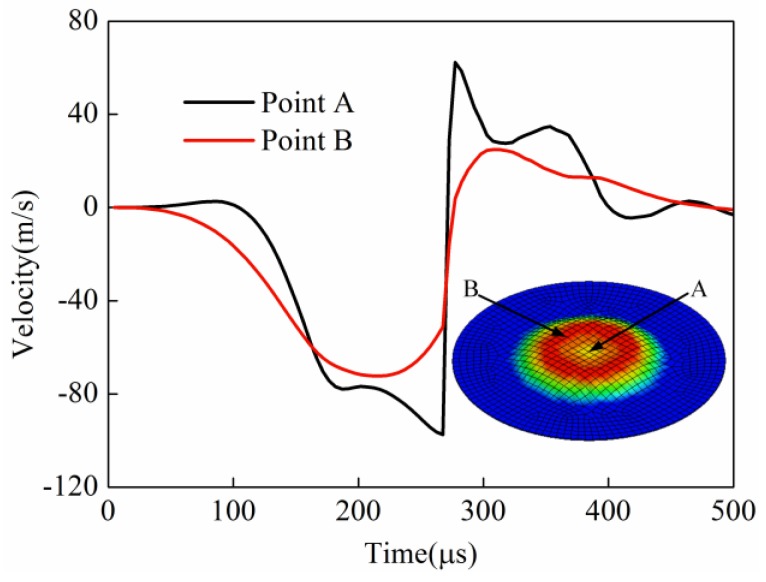
Velocity of point A and point B in direct EMF.

Point A and point B were selected to study the velocity change for direct EMF and indirect EMF. Point A corresponded to sheet center and point B corresponded to the position which was at half of the coil radius. The velocities of point A and point B for direct EMF are shown in Figure 18. It can be seen that in the process of direct EMF, the region near point B was deformed first, then the region near point A was deformed. The speed of point A changed later than at point B, but its speed was greater than point B after 150 μs. When the forming time was 273 μs, the velocity of point A gradually increased to −130 m/s, and the velocity of point B is reached −75 m/s. Point B and point A were successively collided with the die, and the velocities of the two points were reversed respectively. The maximum reverse speed of point A was 40 m/s, and the maximum reverse speed of point B was 20 m/s. The speed of the different points on the sheet were different, which led to different speeds after the collision with the die, so the reverse deformation of the different points after the collision were also different, as shown in Figure 16. After the collision with the die, the speeds of point A and point B were gradually reduced to zero, but the direction of these two points’ velocities were not changed. This is because the sheet was no longer affected by external forces at that time, and it was only under the action of the inertia force. Reverse deformation was then not restrained, which resulted in a final rebound effect.

**Figure 19:**
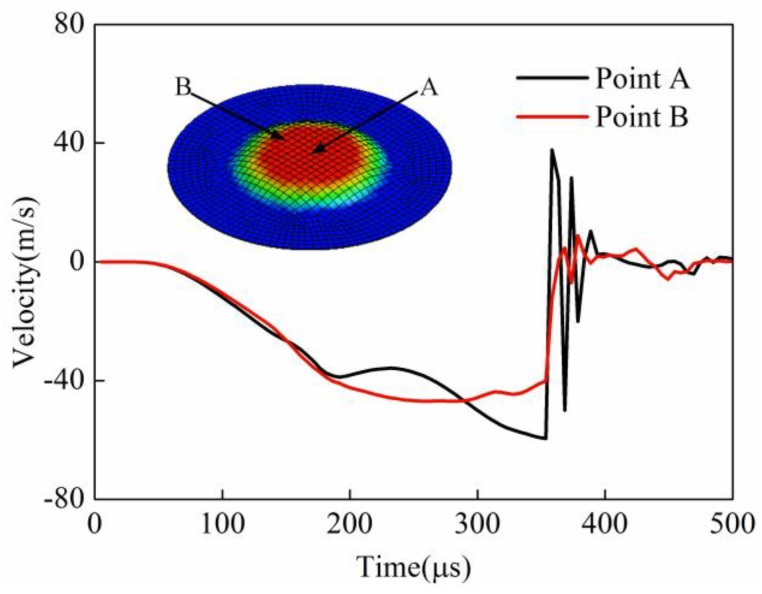
Velocity of point A and point B in indirect EMF.

The velocities of point A and point B for direct EMF with elastic medium are shown in Figure 19. It can be seen that the maximum velocity of point A and point B in indirect EMF were −60 m/s and −40 m/s, which was lower than that of direct EMF. This was because in indirect EMF, part of the whole energy was consumed at deformation of the driver plate and the rubber pad. The energy transferred to the sheet was reduced, which resulted in a reduced kinetic energy of the sheet. When the forming time was 358 μs, point A and point B almost simultaneously collided with the die. It can be found that the direction of the velocity of these two points changed several times, which was markedly different from the direct EMF. This is because in the indirect EMF, the velocity direction of the sheet was changed after collision with the die. However, as the direction of the rubber at the top of the sheet was still downward, the upward movement of the sheet was constrained, so the direction of the sheet was reversed again in a short time. The velocity direction of sheet was changed several times due to the sheet–die interaction and the sheet–rubber interaction. As the movement of the sheet after the collision and the die was suppressed by the rubber, the rebound effect of the workpiece was reduced.

#### 3.2.4. Comparison of Surface Pressure on the Sheet

**Figure 20:**
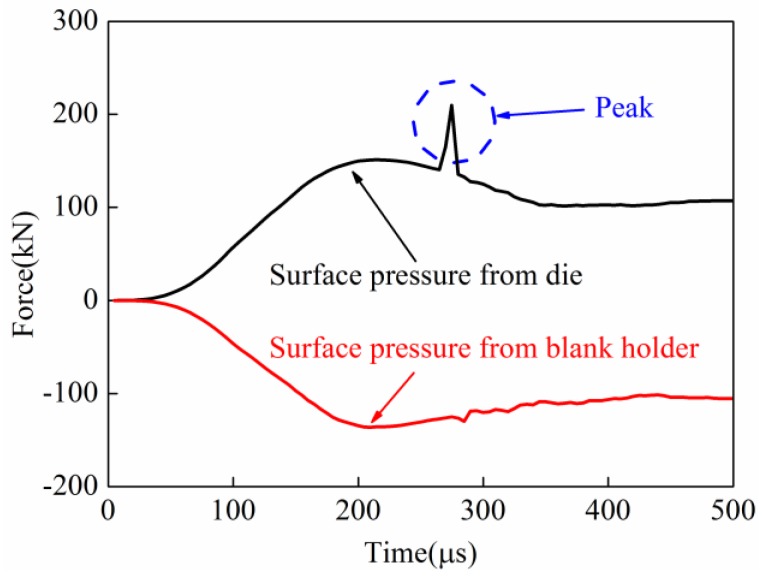
The surface pressure on the sheet in direct EMF.

The surface pressure on the sheet in the process of direct EMF and indirect EMF were compared and analyzed. In the process of direct EMF, the contact force of the upper and lower surfaces of sheet are shown in Figure 20. The lower surface of the sheet was in contact with the die during forming; the upper surface of the sheet was near the coil side. It can be seen that the upper surface of the sheet metal was subjected to pressure from the blank holder, and the lower surface was supported by the female die. The contact force on the upper and lower surface of the sheet was basically equal before 230 μs. The contact force on the lower surface of the sheet was increased quickly to a peak from 230 μs to 245 μs. This was because of the collision between the sheet and die. However, the contact force on the upper surface of the sheet was not increased during this time. When the forming time changed from 245 μs to 265 μs, the contact force between the blank and the die was sharply reduced from 267.1 kN to 131.6 kN. When the forming time was after 265 μs, the contact forces on the upper and lower surfaces of the sheet were nearly the same again. This was because the collision between the sheet and die ended. In conclusion, the contact force from the female die and the contact force from the blank holder during the collision was not balanced in direct EMF, so reverse deformation, which is named the rebound effect, was generated by the deformed workpiece.

**Figure 21:**
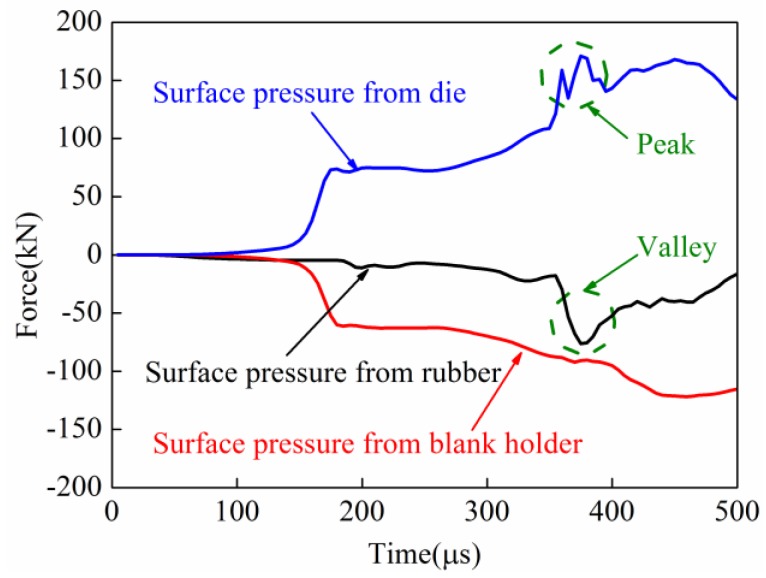
The surface pressure on the sheet in indirect EMF.

In the process of the indirect EMF, the contact force of the upper and lower surfaces of the sheet are shown in Figure 21. It can be seen that in the indirect EMF, the upper surface of the sheet metal was not only subjected to pressure from the pressure from the blank holder, but also under the pressure from the rubber pad. It can be found that there was a peak value of the contact force between the sheet and the female die at 378 μs when the collision occurred, which was the same in direct EMF. But at the same time, there was a valley value of the contact force between the sheet and the rubber pad, which did not exist in direct EMF. It was found that the maximum contact force between sheet and die was 180.3 kN, and the maximum contact force between the sheet and the blank holder was 100.2 kN. At the same time, the contact force between the sheet and the rubber pad was 80.1 kN, which indicated the forces acting on the upper surface and lower surface of the sheet were balanced. It could be concluded that the impact force between sheet and die were balanced by the cushioning force provided by the rubber pad, so the rebound effect was effectively suppressed.

#### 3.2.5. Comparison of Principle Stress of the Sheet’s Center Element

**Figure 22:**
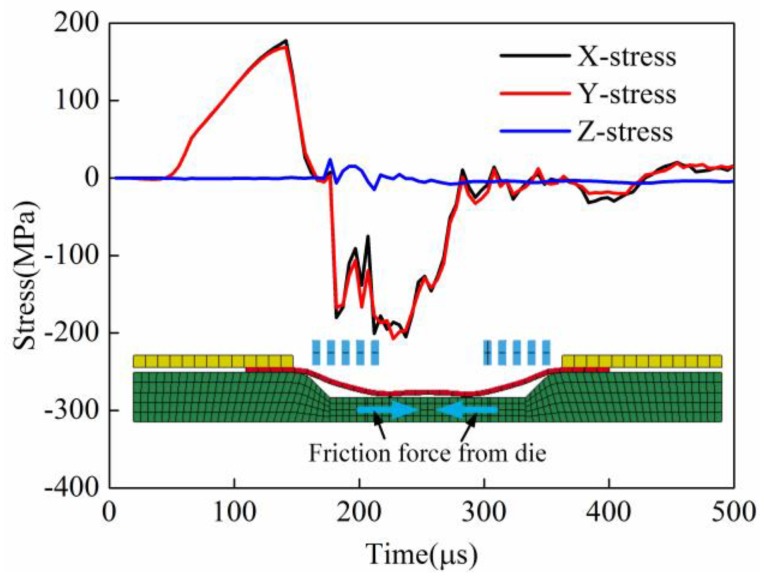
The principle stress of the element at the sheet center in direct EMF.

The element of the sheet center was selected to study the change of the principle stress of direct EMF and indirect EMF. Figure 22 shows the principle stress of the element at the sheet center in direct EMF. It can be seen that the X-direction stress and the Y-direction stress were tensile stresses before 180 μs, this was because the sheet was bulged under the action of the electromagnetic force. When the forming time was 200 μs, the stress state of the X-direction and the Y-direction were changed from tensile stress to compressive stress. The reason may be that when the sheet is in contact with the die, the lower surface of the sheet was subjected to friction force from the die. But at the same time, the upper surface of the sheet was not subjected to any force, as the action of electromagnetic force was ended. Therefore, the center area of the sheet was raised up after the collision between sheet and die. In other words, the friction force on the lower surface of the sheet may be one reason of the rebound effect.

**Figure 23:**
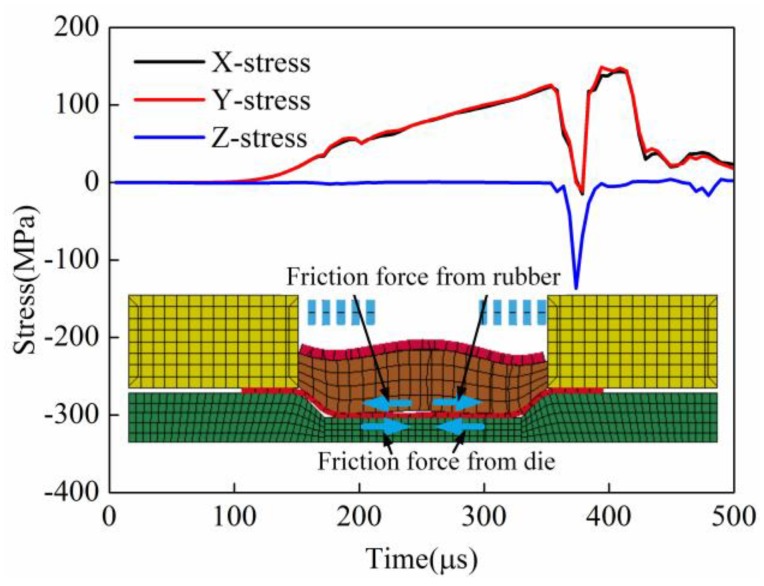
The principle stress of the element at the sheet center in indirect EMF.

Figure 23 shows the principle stress of the element at the sheet center in indirect EMF. It can be seen that the X-direction stress and Y-direction stress were tensile stress, and the stresses increased gradually before 350 μs. This is because that the bottom of the sheet was not in contact with the bottom of the female die, and only the upper surface of the sheet was under the action of friction force from the rubber pad. When the forming time was 350 μs, the stresses decreased rapidly. This is because the bottom of the sheet was in contact with the die, and there was friction force on the lower surface of the sheet, which was the same in direct EMF. But at the same time, it can be found that the directions of the stresses were not changed. The reason maybe that the friction force on upper surface of the sheet was bigger than the friction force on the lower surface of the sheet. In conclusion, the sheet was always under tensile stress state in the indirect EMF, due to the friction force from the rubber. So, the trend of bulging at the sheet center was suppressed; thus the rebound effect of the deformed workpiece was reduced.

#### 3.2.6. Comparison of Energy Change

**Figure 24:**
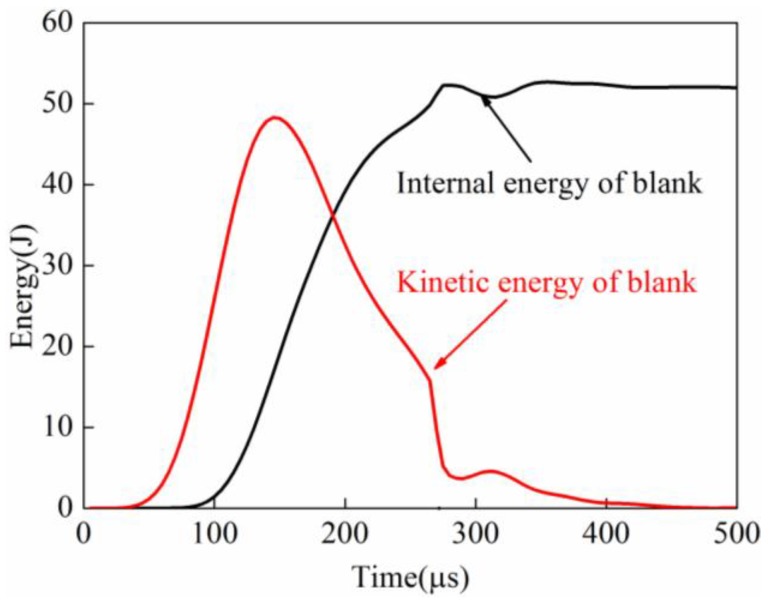
Energy of the sheet in the process of direct EMF.

When the sheet is impacted with the die, the energy would be changing drastically. So, the energy change of the sheet in the process of direct EMF was analyzed. In the process of indirect EMF, the energy changes of the sheet and rubber were analyzed. The energy change of the workpiece in the process of direct EMF is shown in Figure 24. It can be seen that the internal energy of the sheet was gradually increased, and it tended to eventually become stable. The kinetic energy of the sheet is increased first and then decreased, and it reached the maximum value at 150 μs. It was found that when the forming time was 255 μs, the kinetic energy dropped rapidly. This is because the sheet collided with the die at the time, and the majority of kinetic energy is transferred into plastic strain energy of the sheet by the action of friction force from the die. However, after the collision between the sheet and die, the residual kinetic energy of the sheet was still about 10.2 J. The residual kinetic energy of the sheet was not absorbed in a timely manner, which resulted in a reverse deformation of the sheet. When the forming time was 415 μs, the kinetic energy of the sheet was reduced to zero. So, the total time from the time after the collision to the time when kinetic energy of the sheet was completely to zero, was 160 μs. In conclusion, the rebound effect occurred because the residual kinetic energy of the sheet after the collision with the die could not be absorbed in time.

**Figure 25:**
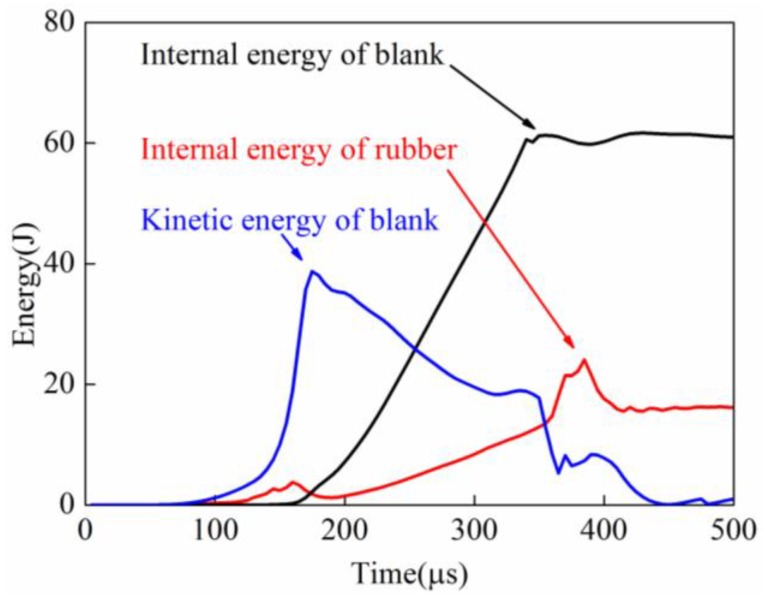
Energy change in indirect EMF.

Figure 25 shows the energy change of sheet and rubber in indirect EMF. It can be seen that the kinetic energy of the sheet was increased first, and then it decreased, and the kinetic energy reached the maximum value at 174 μs. When the forming time is 367.5 μs, the kinetic energy of the sheet is rapidly decreased. The residual kinetic energy after collision was about 3.5 J, it was smaller than that of direct EMF. This is because that part of the kinetic energy was transferred to the elastic energy of the rubber. When the forming time was 446 μs, the kinetic energy of the sheet was reduced to zero. So, the total time from the time after collision to the time when the kinetic energy of the sheet was reduced completely to zero was 78 μs. The velocity attenuation rate of the sheet is faster than that in direct EMF. It can also be found that there is a wave crest of the rubber internal energy, which indicated that part of the residual kinetic energy of sheet was absorbed by the rubber pad. So as a result, the rebound effect in indirect EMF is reduced.

## 4. Conclusions

Aiming at reducing the rebound effect caused by high speed collision between the workpiece and die in direct EMF, this paper studied the factors influencing the fittability of the workpiece in indirect EMF, forming the process of direct EMF and indirect EMF are compared and analyzed, and the mechanisms of suppressing the rebound effect by indirect EMF are revealed. The conclusions are as follows:

(1) The finite element models of direct EMF and indirect EMF can predict the experimental results effectively. The rebound effect can be effectively restrained in indirect EMF with elastic medium, and the fittability of workpiece is improved compared with direct EMF. 

(2) For indirect EMF, with the increase of discharging voltage or the increasing of rubber thickness, the fittability of workpiece is increased firstly and then decreased. When the thickness of the driver plate is equal to the skin depth of the driver plate, the fittability of deformed workpiece is the best. In this paper, the optimal process parameters for indirect EMF are as follows: the discharging voltage is 10 kV, the rubber thickness is 20 mm, and the thickness of the driver plate is 2 mm.

(3) The velocity at different points of the sheet is different in the direct EMF, so the reverse deformation of different points after collision with the die are different. The velocity at different points of the sheet are nearly the same in indirect EMF, and the reverse deformation is nearly zero at a proper forming condition.

(4) The rebound effect of the workpiece in indirect EMF can be suppressed in the indirect EMF because: the movement of the sheet after the collision and the die is suppressed by the sheet–rubber interaction; the impact force caused by the collision between sheet and die is balanced by the pressure provided by the rubber; the sheet is always under tensile stress state due to the friction force provided by rubber; the remaining kinetic energy of sheet after the collision with the die is absorbed by rubber.

## Figures and Tables

**Table 1:** Influence factors and levels table of indirect EMF.

Levels	Discharging Voltage (kV)	Thickness of Driver Plate (mm)	Thickness of Rubber (mm)
1	6	1	10
2	8	2	20
3	10	3	30
4	12	4	40

**Table 2:** Orthogonal experimental table of indirect EMF.

NO	Discharging Voltage (kV)	Thickness of Driver Plate (mm)	Thickness of Rubber (mm)	η(%)
1	6	1	10	77.1
2	6	2	20	70.7
3	6	3	30	36.6
4	6	4	40	36.5
5	8	1	20	79.9
6	8	2	10	86.6
7	8	3	40	60.3
8	8	4	30	36.6
9	10	1	30	86.8
10	10	2	40	82.2
11	10	3	10	89.8
12	10	4	20	93.7
13	12	1	40	86.1
14	12	2	30	90.3
15	12	3	20	93.6
16	12	4	10	92.8
